# Transduction and Oncolytic Profile of a Potent Replication-Competent Adenovirus 11p Vector (RCAd11pGFP) in Colon Carcinoma Cells

**DOI:** 10.1371/journal.pone.0017532

**Published:** 2011-03-24

**Authors:** Jim Silver, Ya-Fang Mei

**Affiliations:** Department of Clinical Microbiology/Virology, Umea University, Umeå, Sweden; University of Hong Kong, Hong Kong

## Abstract

Replication-competent adenovirus type 5 (Ad5) vectors promise to be more efficient gene delivery vehicles than their replication-deficient counterparts, and chimeric Ad5 vectors that are capable of targeting CD46 are more effective than Ad5 vectors with native fibers. Although several strategies have been used to improve gene transduction and oncolysis, either by modifying their tropism or enhancing their replication capacity, some tumor cells are still relatively refractory to infection by chimeric Ad5. The oncolytic effects of the vectors are apparent in certain tumors but not in others. Here, we report the biological and oncolytic profiles of a replication-competent adenovirus 11p vector (RCAd11pGFP) in colon carcinoma cells. CD46 was abundantly expressed in all cells studied; however, the transduction efficiency of RCAd11pGFP varied. RCAd11pGFP efficiently transduced HT-29, HCT-8, and LS174T cells, but it transduced T84 cells, derived from a colon cancer metastasis in the lung, less efficiently. Interestingly, RCAd11p replicated more rapidly in the T84 cells than in HCT-8 and LS174T cells and as rapidly as in HT-29 cells. Cell toxicity and proliferation assays indicated that RCAd11pGFP had the highest cell-killing activities in HT29 and T84 cells, the latter of which also expressed the highest levels of glycoproteins of the carcinoma embryonic antigen (CEA) family. *In vivo* experiments showed significant growth inhibition of T84 and HT-29 tumors in xenograft mice treated with either RCAd11pGFP or Ad11pwt compared to untreated controls. Thus, RCAd11pGFP has a potent cytotoxic effect on colon carcinoma cells.

## Introduction

Colon carcinoma is the second most common malignancy in humans after lung cancer. The five-year survival rate is about 50% [1]. Survival rates are directly linked to how early the cancer is diagnosed, and they have not improved significantly over the past 40 years, despite advances in treatment [2]. More than half of the patients in remission suffer from a recurrence of the original cancer and many eventually die from it. Gene therapy based on adenovirus vectors may represent an alternative to conventional cancer treatment and vaccination [3], [4], [5]. However, because the early region 1 (E1) gene has been deleted from these virus genomes to generate replication-defective vector and to increase their capacity to carry foreign genes, the ability of these defective vectors to infect tumor cells and spread to adjacent cells is limited[6], [7]. Moreover, most adult patients are already immune to the commonly used adenovirus serotype, Ad5; as a consequence, a high dose of vector is required for effective treatment [8]. Strategies to circumvent these limitations have involved the use of other adenovirus types that have a better capacity to replicate *in vivo*.

There are 53 serotypes of human adenoviruses, which have been grouped into seven species (designated A to G). Species B adenoviruses have been classified further into two subspecies, B:1 and B:2. Ad11, Ad34 and Ad35, all of which are subspecies of B:2 adenoviruses, were isolated from immunosuppressed patients and can cause human urinary tract infections. Ad11 has been classified further into two genome types, Ad11p and Ad11a, which cause urinary and respiratory infections, respectively [9]. In our previous studies, Ad11p, Ad11a, and Ad35 showed varying patterns of binding kinetics to human cell lines of different origins, where Ad11p and Ad35 (with tropism to the urinary tract) had a higher binding affinity than Ad11a (with respiratory tropism) [10]. Species B adenoviruses use CD46 and, evidently, CD80 and CD86 as primary receptors [11], [12]. The most commonly used vector, Ad5 of species C, causes mild respiratory infection in children. Ad41 belongs to species F and is associated with infantile diarrhea. The fiber knobs of Ad5 and Ad41 viruses bind primarily to coxsackievirus and adenovirus receptor (CAR). Many tumor cells express relatively low levels of CAR, which essentially renders the tumor cell resistant to Ad5 infection. The major capsid component, the hexon, is also involved in hepatocyte infection [13].

Adenoviruses with enhanced transduction efficiency have been generated using chimeric fiber knobs or the insertion of peptides into the knob region. This strategy is based on CAR. It is the receptor of most human adenoviruses, except species B, and it is downregulated on the surface of tumor cells. In contrast, species B adenoviruses use the CD46 molecule, which is also abundantly expressed on most human cells and is upregulated in carcinoma cells[14] [15]. Interestingly, “desmoglein-2 (DSG-2)”, the newly defined receptor for Ad3, Ad7, Ad11p and Ad14 are also overexpressed on a series of epithelial malignancies [16]. Ad5 vectors with modified fiber knobs have allowed for great improvements in the efficiency of gene transduction[17] [18] [19] [20]. Defective adenovirus vectors from species B adenoviruses have been reported[21] [22] [23], but a replication-competent vector to improve virus replication, cell lysis, and virus release has not been identified. There is a lack of species B vectors that carry quantifiable marker genes and still have the capacity to replicate as well as the wild-type virus. A precise evaluation of the oncolytic vectors in different tumor cells remains to be addressed.

In this study, we took a different approach by analyzing the infectivity of adenoviruses of species B, C, and F in colon carcinoma cell lines. We then focused on the initial transduction, cell toxicity, proliferation, and oncolytic effect of a replication-competent adenovirus 11 prototype expressing green fluorescence protein (RCAd11pGFP). The tumor-specific killing effect mediated by RCAd11pGFP was further improved in xenograft nude mouse models.

## Results

### Adenovirus species differ in their ability to infect colon carcinoma cell lines

**Figure 1:**
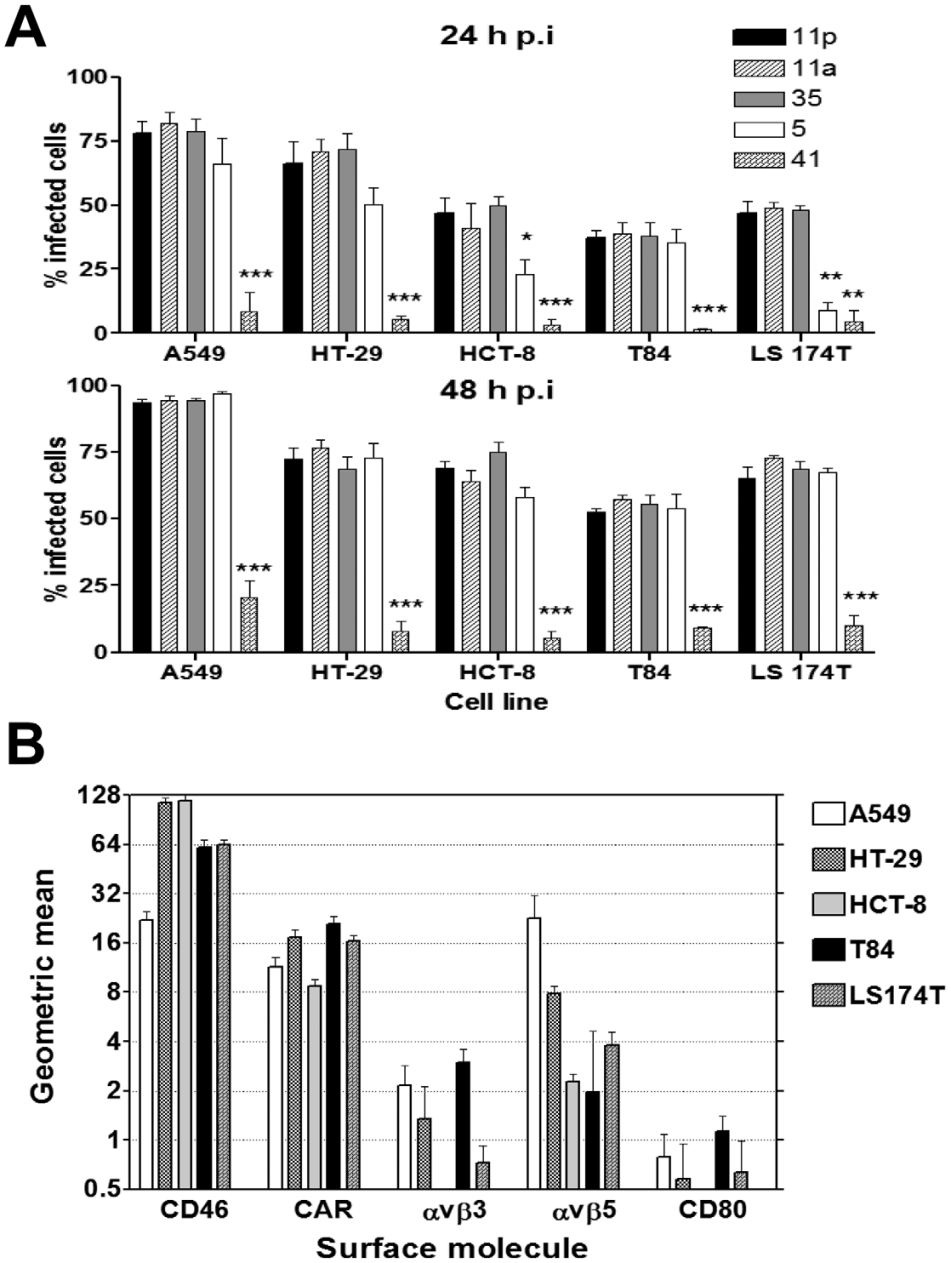
Permissiveness of the carcinoma cells to the adenovirus species and relevant cell-surface molecules on the colon carcinoma cells. (A) Four different colon carcinoma cell lines and A549 cells were infected with two CAR-binding genome types (Ad5 and Ad41p), with two CD46-binding genome types (Ad11p and Ad35), and with one genome type that binds to an unidentified receptor (Ad11a). The permissiveness of infected cells was measured by staining the cells for hexon production 24 and 48 h p.i., followed by flow-cytometric analysis. The virus concentration used was 3,600 vp per cell. The results are presented as the mean ± SEM of at least three independent experiments. Significant differences from Ad11p virus are indicated above each bar: * *P*<0.05; **P<0.01; ***P<0.001. (B) The expression of relevant cell-surface molecules on colon carcinoma cell lines was assessed by FACS. The expression of human CD46, CAR, integrin αυβ3, integrin αυβ5, and CD80 on colon carcinoma cell lines was determined by flow cytometry using monoclonal antibodies to cell-surface molecules.

The permissiveness of the carcinoma cells to the different adenovirus species was evaluated by measuring the proportion of cells expressing viral hexon protein at 24 and 48 h p.i. Species B adenoviruses Ad11p, Ad11a, and Ad35 infected HT-29, T84, LS174T and HCT-8 colon carcinoma cell lines, with 35–80% of the cell infection at 24 h (Figure 1A). In contrast, Ad5 (species C) showed a lower degree of infectivity of the LS174T and HCT-8 cell lines compared to species B adenoviruses 24 h p.i. This difference disappeared by 48 h p.i. All colon carcinoma cell lines and the lung carcinoma cell line A549 (used as a control) were inefficiently infected by the species F adenovirus Ad41; ≤20% of cells were positive for the expression of Ad41 hexon at 48 h p.i.

### Expression of the relevant receptor molecules varies in different colon carcinoma cell lines

We studied the distribution of cell-surface molecules that function as adenovirus receptors or tumor antigens in colon carcinoma cancer cells. CAR and CD46 serve as receptors for Ad5 and Ad11, respectively[24] [25] [26]. Flow-cytometric analysis of the immunofluorescence staining showed that in general, all colon carcinoma cell lines studied had a higher level of CD46 expression than A549 cells, but expression levels of the internalization receptors, integrins αυβ3 and αυβ5, were lower in comparison to A549 cells (Figure 1B). In T84, HT-29, and LS174T cells, the geometric mean values of CAR expression were 22.3, 16.8, and 16.4, respectively, whereas A549 and HCT-8 cells expressed lower levels of CAR, with geometric mean values of 11.6 and 8.7, respectively. CD46 was abundant on HT-29 and HCT-8 cells, with geometric mean values of 115.8 and 118, respectively, which was more than four times higher than the level in A549 cells (22.2). An intermediate level of expression of CD46 (Figure 1B) was observed in T84 and LS174T cells, with geometric mean values of 61.5 and 63.7, respectively.

We compared the amounts of integrins αvβ3 and αvβ5 and also of CD80 expressed on the cell surfaces of the colon carcinoma cell lines with those on A549 cells. Integrin αvβ5 was expressed at a high level on A549 cells; the geometric mean reached 21.4, which was greater than the expression in all colon carcinoma cells. Integrin αvβ3 was also detected at a relatively lower level than integrin αvβ5 on all cell surfaces. The expression level of αvβ5 in A549 cells was higher than in all other cell lines studied, in which the geometric mean was only 2.3. In the cells studied, CD80 expression was not significantly above the detection limit, i.e., the geometric mean for colon carcinoma cells was less than 1. It was difficult to assess whether CD80 also acts as a receptor for Ad11 on colon carcinoma cells, as its expression was undetectable (Figure 1B).

### RCAd11p efficiently transduces the GFP gene into colon carcinoma cells

**Figure 2:**
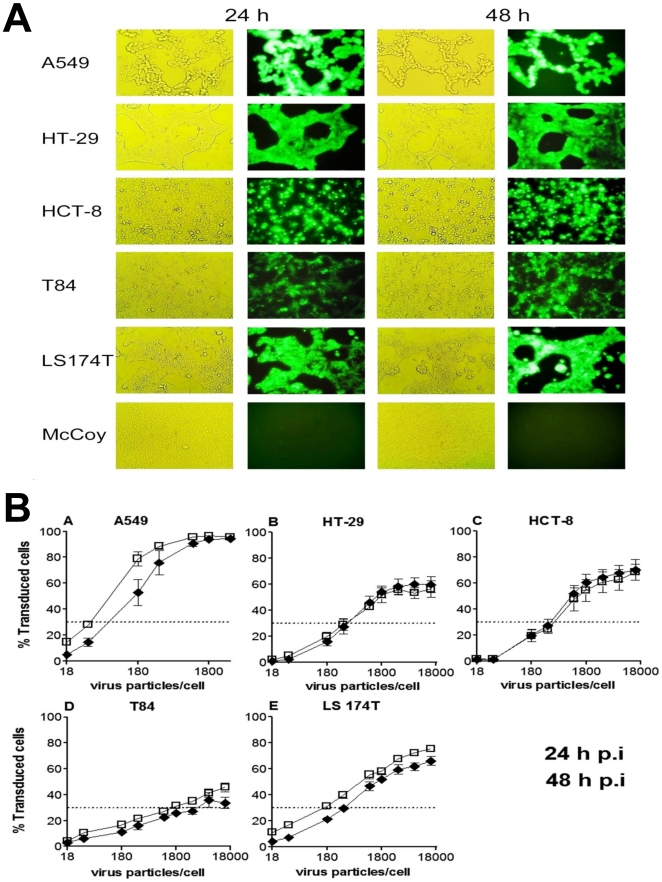
Transduction efficiency of RCAd11pGFP in human colon carcinoma cells. (A) Transduction and cytopathic effects assay with fluorescence microscopy and phase contrast light microscopy. Cells were transduced with RCAd11pGFP at 3,600 vp per cell. At 24 and 48 h p.i., cells expressing GFP and cells showing cytopathic effects were detected by fluorescence microscopy and light microscopy (200× magnification). (B). Transduction efficiency of RCAd11p in four human colon carcinoma cell lines and control A549 and McCoy cells. Cells were infected with RCAd11pGFP at 18; 36; 180; 360; 1,100; 1,800; 3,600; 7,200; or 14,400 vp per cell, whereas A549 cells were infected with up to 3,600 vp per cell. The percentage of cells expressing GFP was assessed by flow-cytometric analysis at 24 and 48 h. p.i. The results presented here are from at least three independent experiments. Error bars represent SEM.

To determine whether there might be a correlation between the amount of cell-surface molecules that can serve as primary or secondary receptors and the efficiency of gene transduction, we performed kinetic experiments to quantify the expression of GFP in various colorectal cancer cells. Serially diluted RCAd11pGFP was introduced into the target cells. Twenty-four and 48 h after infection, GFP expression was determined by flow cytometry. RCAd11pGFP was clearly capable of infecting all the cell lines tested (Figure 2A and 2B), but there was a significant difference in transduction efficiency between cells infected with different doses of RCAd11pGFP (0.36 or 3,600 vp per cell). The number of GFP-positive cells at 24 h p.i. was similar in HT-29, HCT-8, and LS174T cells (in the range of 25–30% positive cells) (Figure 2B) infected with 0.1 pg of RCAd11pGFP per cell, which was equivalent to a multiplicity of infection (MOI) of 5 (360 vp/cell). In contrast, only 16% of T84 cells were GFP-positive under the same conditions. With the high dose of 1 pg of RCAd11pGFP per cell (MOI of 50; 3,600 vp per cell), the proportion of GFP-positive cells increased to 57.8%, 64.3%, and 59.2% for HT-29, HCT-8, and LS174T cells, respectively. With a high dose of RCAd11pGFP (1 pg/cell), only 27.4% of the T84 cells were positive. Thus, the relationship between the vector concentration and transduction efficiency suggests that a ten-fold greater dose of RCAd11pGFP results in approximately a two-fold increased transduction. Thus, RCAd11pGFP transduced colon carcinoma cells in a dose-dependent manner. Also, various amounts of RCAd11pGFP were required to achieve a level of transduction of 50% of cells expressing GFP. As shown in Table 1, 1,100, 1,400, 1,400, and 7,000 vp per cell were required for this level of expression in HCT-8, HT-29, LS174T, and T84 cells, respectively (Figure 2A and 2B).

**Table 1:** Summary of receptor and CEA molecules, GFP expression and oncolytic effect mediated by RCAd11pGFP.

Cell type	CD46	ανβ3	ανβ5	CEA	GFP expression		Oncolytic effect		Ratio (fold)***
					24 h	48 h	6 day	12 day	
A549	22.2	2.3	21.4	83	180	110	3.6	<0.36	500
HT-29	115.8	1.7	7.74	204.6	1400	1800	20	0.36	5000
HCT-8	118	0.3	1.5	123	1100	1100	360	36	388
T84	61.5	1.37	2.68	769.8	7000*	7000**	36	3.6	1944
LS174T	63.7	0.73	3.39	89.5	1400	700	36	>3.6	30.6

**Note**: Geometric mean was used to compare the amounts of CD46, avb3, avb5, and CEA molecules expressed on the different cell surfaces.

*: 7,000 vp/cell gave 36% GFP-expressing cells.

**: 7,000 vp/cell gave 41% GFP-expressing cells.

***: Ratio (fold) of cell lysis to transduction means the ratio of the cytolytic effect of the amount of RCAd11pGFP virus particles (vp) required to obtain 50% cell lysis at 12 days p.i. to the transduction efficiency of the vector (vp) required to obtain 50% GFP-expressing cells at 24 h p.i. This ratio was compared in each colon carcinoma cell line. The results are presented as the mean value of three independent experiments. A549 cells were used as the control cell line.

As stated above, the level of expression of CD46 on A549 cells was lower than that on HT-29 and HCT-8 cells. There was no correlation between cell transduction and the level of CD46 on the colon cancer cells tested. In contrast, αvβ3 and αvβ5 integrins probably played a secondary but critical role in RCAd11pGFP transduction because A549, HT-29, and LS174T cells all expressed a relative high amount of αvβ5 integrin accompanied by high transduction levels. It can be concluded from the above results that viral transduction depends not only on the density of CD46 on the colon carcinoma cell surface but also perhaps on integrin expression. Of the cell lines tested, A549 cells, with high expressions of αv-integrin, were the most sensitive to infection by RCAd11pGFP, and T84 cells with low expression of αv-integrin were the least sensitive.

### The cytotoxic effect of RCAd11pGFP is more obvious in HT-29 and T84 cells than in HCT-8 and LS174T cells

**Figure 3:**
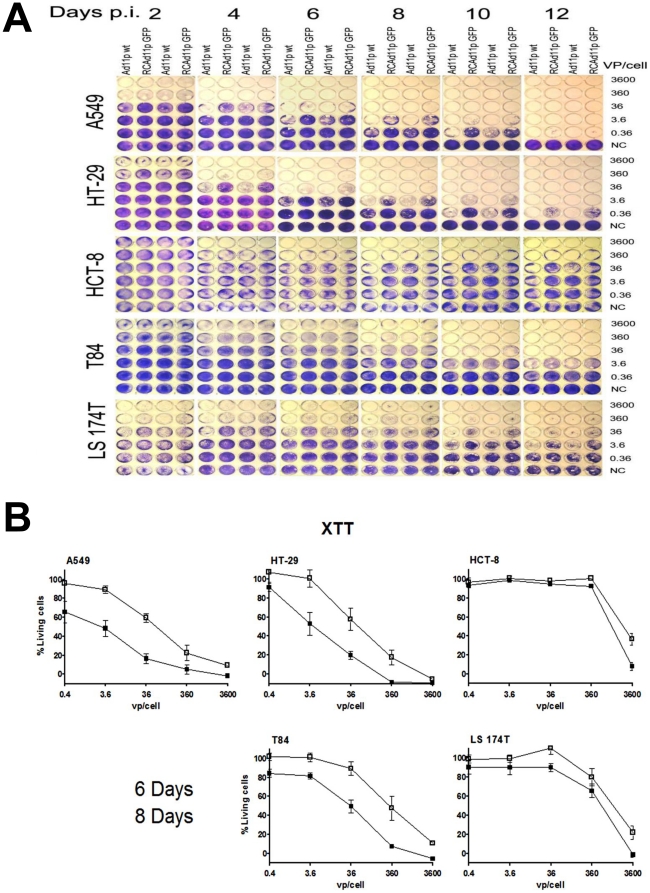
Comparison of the oncolytic and cytolytic effects of RCAd11pGFP and Ad11pwt on colon carcinoma cells. (A) Oncolytic effects. Monolayers of HT-29, HCT-8, T84, LS174T, and the control A549 cells were infected separately with serial dilutions of Ad11pwt and RCAd11pGFP. The virus concentration is shown on the right. Every other day, cells were washed and stained with crystal violet. RCAd11pGFP and Ad11pwt spread very efficiently from cell to cell in HT-29 and T84 cell lines but less efficiently in LS174T and HCT-8 cells. One representative experiment out of three is shown. (B) Cell toxicity assay. Monolayers of HT-29, HCT-8, T84, LS174T, and control A549 cells were infected with serial dilutions of RCAd11pGFP. At 6 and 8 days p.i., XTT was added to the cells and incubation was continued for 2 h, and then the number of living cells was assessed in a plate reader as described in Materials and Methods. The result represents the mean of three independent experiments. Error bars represent SEM.

To evaluate the oncolytic activity of RCAd11pGFP, we performed toxicity assays on colon carcinoma cells and A549 cells. As shown in Figure 3A and Table 1, cells in 24-well plates were infected with 10-fold dilutions of RCAd11pGFP and wild-type Ad11p (Ad11pwt) starting at 3,600 vp per cell. As determined at 2-day intervals, cells infected with the high MOI of 50 (3,600 vp per cell) were rapidly destroyed. However, in cells infected with a low MOI of 0.005 (0.36 vp per cell), it took the virus one or more cycles of infection to have a detectable cytopathic effect (CPE). When the oncolysis of RCAd11pGFP and Ad11pwt was compared, no great difference was observed in any of the colon carcinoma cell lines studied (Figure 3A). RCAd11pGFP replicated 100 times more efficiently in HT-29 cells than in HCT-8 cells, whereas oncolysis was 10 times more efficient in T84 cells than in HCT-8 and LS174T cells (i.e., requiring 3.6 vp per cell vs. 36 vp per cell), even though T84 cells showed less GFP expression than HCT-8 and LS174T cells 24 h p.i. These results indicate that the replication cycle of RCAd11pGFP vector differed greatly in a cell-specific fashion. Consequently, RCAd11pGFP replicated 10 and100 times more efficiently in T84 and HT-29 cells, respectively, than in HCT-8 cells.

### RCAd11pGFP causes significantly cytolytic effect on HT-29 and T84 cells

To study the effect of RCAd11pGFP on cell viability and its cell-killing effect, and also to confirm the toxicity results, the colon carcinoma cell lines and control A549 cells were infected with the virus concentrations indicated in Figure 3B. No distinct change in viability was found in HCT-8 cells at the lower moi 3.6, 36 and 360 vp/cell but a 60 to 80% of viability reduced at the highest moi (3600 vp/cell) at six or eight days p.i; however, the viability of other colon carcinoma cells was clearly reduced between eight and six days p.i. The viability of HT-29 cells was reduced to 60% when the cells were infected with 36 vp per cell for 6 days, and it was further reduced by 20% at 8 days p.i. The same effect was also seen in A549 cells. With the same infectious dose, the viability of T84 cells was 25–30% higher than that of HT-29 cells but 40% lower than that for HCT-8 and LS174T cells. In general, the dose of RCAd11pGFP required to obtain 50% viability differed greatly, as 3.6 vp per cell were required for A549 and HT-29 cells, 36 vp per cell were needed for T84 cells, and 360 or more vp per cell were needed for HCT-8 and LS174T cells. Consequently, the results of the toxicity assays indicate that HT-29 and T84 cells are killed more efficiently than HCT-8 or LS174T cells.

### RCAd11pGFP shows a higher oncolytic effect in HT-29 and T84 cells than in HCT-8 and LS174T cells

GFP expression and cell lysis mediated by RCAd11pGFP varied substantially in the various colon carcinoma cell lines. To determine whether the tumor cells also affect virus replication, we compared the infectious doses (180 vp per cell for A549; 1,140 vp per cell for HT-29; and >7,000 vp per cell for T84 cells) used in the transduction that gave 50% of transfected cells, with the oncolytic doses (0.36 vp per cell for A549 and HT-29, and 3.6 for T84 cells) used in the toxicity assay that caused 50% cell lysis (LD_50_).

To measure the oncolytic effect of RCAd11pGFP on the colon cancer cells, the ratio of oncolytic effect to transduction efficiency was calculated for each cell line (the amount of RCAd11pGFP virus particles required to cause 50% cell lysis at 12 days p.i. versus the number of virus particles required to obtain 50% GFP-expressing cells at 24 h p.i.). The oncolytic effect was increased 5,000-fold in HT-29 cells; 1,944-fold in T84 cells; and 388-fold in LS174T cells compared to only 30-fold in HCT-8 cells (Table 1). Although GFP expression in HCT-8 cells at 24 h p.i. was greatest compared to other colon carcinoma cell lines, the lowest toxicity was observed after 12 days p.i. Conversely, the lowest level of GFP expression but a higher level of toxicity was observed in T84 cells at 12 days p.i. Both T84 and HT-29 cells showed high tumorigenesis in nude mice.

### HT-29 and T84 cells show higher expression of CEA family molecules than HCT-8 and LS174T cells

**Figure 4:**
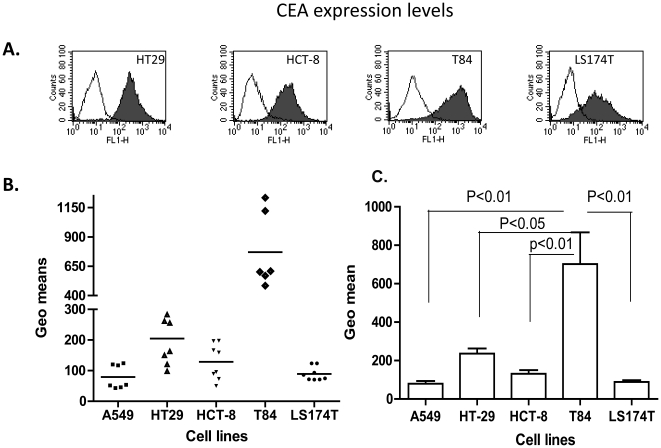
Measurement of the amount of CEA antigen by flow cytometry. An anti-CEA polyclonal antibody was used to detect the expression levels of CEA family molecules in different colon carcinoma cell lines. (A). Original FACS graphs for the CEA family molecule. (B) Geometric mean values for CEA expression on each cell surface were measured several times. T84 cells produced the most CEA, HT-29 cells expressed CEA at medium levels, and both HCT-8 and LS174T cells expressed the lowest amount of CEA family molecules. (C). Statistical analysis (*t*-test) of CEA expression levels in colon cancer cell lines.

Carcinoembryonic antigen (CEA) is the prototypic member of a highly similar group of cell-surface glycoproteins. The CEA family has been used as a prognostic marker for colorectal cancers [27]. A rabbit polyclonal anti-CEA antibody (provided by Prof. Sten Hammarström of the Department of Clinical Microbiology, Umeå University) was expected to cross-react with CEACAM5 and CEACAM6. We found that the expression of CEA family glycoproteins showed striking variation in the cell lines tested; all colon carcinoma cells showed higher expressions of these molecules than A549 cells. Interestingly, a quantitative analysis of the tumor marker proteins showed that CEA molecules were more abundantly expressed in T84 and HT-29 cells than in the other two colon carcinoma cell lines (Table 1, Figure 4). T84 cells are derived from metastatic colon carcinoma cells taken from the lung, and they expressed the highest amounts of CEA family molecules, with 8.5-fold and 9-fold greater expression than in LS174T and A549 cells, respectively. The HT-29 cell line has the second highest expression of CEA and is the most tumorigenic colon carcinoma cell line studied, and the level of expression of CEA family molecules in HT-29 cells was 2.47-fold higher than in A549 cells. CEA family expression in HCT-8 cells was only 1.3-fold and 1.5-fold higher than in LS174T and A549 cells, respectively. In the four colon carcinoma cell lines, the greatest to the least expression of CEA family molecules was in the following order: T84, HT-29, HCT-8, and LS174T (Figure 4A, 4B and 4C). These findings indicate that infection with RCAd11pGFP results in selective replication in colon carcinoma cells, which express high levels of CEACAM5 and CEACAM6.

### Oncolytic activity of the RCAd11p vector in animal models

**Figure 5:**
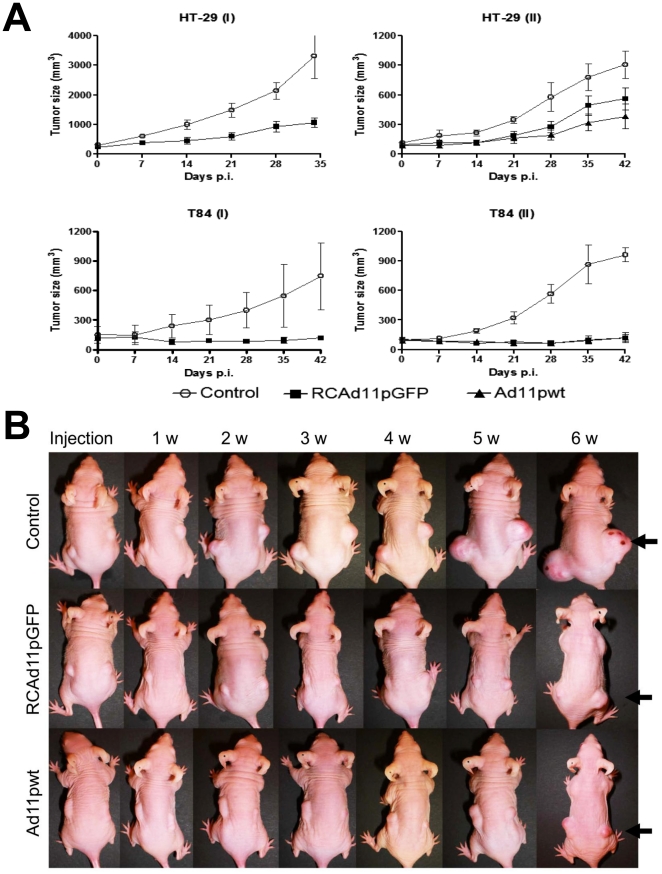
The antitumor effect of RCAd11pGFP and Ad11pwt viruses on colon tumors in BALB/c mice. (A) Summary of the oncolytic effect mediated by RCAd11pGFP and Ad11pwt on HT-29 tumors and T84 tumors in xenograft mice. HT-29 cells are derived from colorectal adenocarcinoma and T84 cells are derived from a lung-derived metastasis of a colorectal adenocarcinoma, T84 cells (10^7^/tumor) or H-29 cells (5×10^6^) in 0.2 ml PBS were subcutaneously transplanted into the left and right flank region of BALB/c nude mice, and control mice were injected with PBS alone. The colon tumors grew to at least 75 mm^3^ about 2 weeks after the injection of cells, and 50 µg of either RCAd11p or Ad11pwt were injected into the tumor. The tumor volumes were recorded weekly. The mice were sacrificed approximately six weeks after injection of the virus or PBS. (B) Photographs of T84 tumor inhibition by RCAd11p and Ad11pwt were recorded weekly. Black arrows indicate T84 tumors.

We examined the ability of RCAd11pGFP to inhibit the growth of pre-established human tumor xenografts in Balb/C nude mice. Tumors were formed by injecting HT-29 and T84 cells s.c. into both hind flanks of mice When the tumor reached approximately 75 mm^3^_,_ the tumor were injected once with 50 µg of RCAd11pGFP or Ad11pwt viruses by intratumoral injection. Tumor growth was measured weekly and for total of 6 weeks p.i. No significant toxicity was observed in these mice. The growth of two colon cancer xenografts was suppressed effectively by both RCAd11pGFP and Ad11pwt viruses (Figure 5A). At day 32 of the first run, untreated mice carrying HT-29 tumors had to be killed due to uncontrolled tumor growth. Over the course of the experiment, untreated HT-29 tumors grew 4-5-fold at 3 w p.i. and 11-fold at 6 w p.i. in comparison of its initial tumor volume, whereas the RCAd11pGFP-injected tumor grew only approximately 1.7–2.4 fold at 3 w and 4.2 fold at 6 w p.i. In the T84 tumor xenograft, we observed an even more significant difference of RCAd11pGFP versus mock (Figure 5B), the tumors injected with the vector were suppressed to 0.8 fold 3 w p.i. and grew approximately 1.3 fold 6 w p.i. In contrast, untreated tumors rapidly grew 2.1 to 3.9 fold 3 w p.i. and 5.4 to 9.2 fold 6 w p.i.

## Discussion

Three main points summarize the results presented here. 1) There was no strong correlation between RCAd11p-mediated transduction and the amount of CD46 expressed on the surface of colon carcinoma cells lines. There was an excess of CD46 molecules on most tumor cells. 2) There was a correlation between the degree of expression of CEA family molecules on colon tumor cells and the oncolytic effects *in vivo*. 3) HT-29 and T84 cells showed the strongest oncolytic effects after infection with RCAd11pGFP, and HCT-8 cells showed the weakest effects. Overall, these results suggest that the extent of adenovirus-induced oncolytic effects depends on the type of tumor cell.

Substantial differences in infectivity were observed between species B, C, and F adenoviruses. The hexon proteins of the species B adenoviruses Ad11p, Ad11a, and Ad35 were all efficiently expressed in the different human colon carcinoma cells within 24 h of infection, and increased expression was noted after 48 h (Figure 1A). The infection pattern of Ad5, which binds to CAR, varied between different cell lines: after 24 h, Ad5 infection was already on par with species B adenovirus infection in HT-29 and T84 cells, whereas 48 h was required for the Ad5 infection to reach the level achieved by species B adenoviruses in HCT-8 and LS174T cells. Ad41 infected all colon carcinoma cell lines inefficiently. These findings support the use of species B adenoviruses as vectors for the infection of colon carcinoma cells.

CD46 and integrins on the cell surface are important for binding, uptake, and infection by species B adenoviruses [28]. There is little information on whether the level of expression of these molecules affects transduction mediated by RCAd11pGFP. CD46 was expressed most on HT-29 and HCT-8 cells and was moderately expressed on T84 and LS174T cells. Ad11p uses CD46 as its primary receptor and infects cells by a route that is independent of CAR [25]. A number of other cellular receptors most likely also contribute to the transduction efficiency [29], [30]. Screening for cell-surface receptors with monoclonal antibodies has shown that the integrins αvβ3 and αvβ5 are also important for infection [28]. All colon carcinoma cells tested in the present study expressed higher amounts of CD46 than did A549 cells. A549 cells expressed the lowest level of CD46 but the highest amounts of integrins αvβ3 and αvβ5. A549 cells were the most permissive for RCAd11pGFP infection, as they showed the highest level of GFP expression at 24 h p.i. (Figure 2A and 2B). Integrin αvβ5 was detected at a low level on HT-29 and LS174T cells and was not clearly detectable on HCT-8 and T84 cells. Taken together, these results also indicate that the amounts of CD46 molecules, αvβ3 and αvβ5 integrins on the different colon carcinoma cells play decisively important role for RCAd11pGFP to enable internalization.

One of the major differences in the approach using oncolytic RCAd11pGFP compared to traditional, replication-defective Ad5 vectors is that RCAd11pGFP can provide an opportunity to measure the transduction efficiency, and it can also replicate and produce progeny viruses. We monitored the transduction and replication capacity of the RCAd11pGFP vector in colon carcinoma cells. GFP expression indicates the transduction efficiency in the nucleus, whereas the interaction between the vector and the nuclear components of the host cell causes an oncolytic effect. However, the extent of oncolysis varied substantially between the different colon carcinoma cells; there was no direct correlation between transduction efficiency and oncolytic capacity. Transduction in LS174T and HCT-8 cells was highly efficient, whereas cell killing (LD_50_) or oncolysis in these cells was 10- to 100-fold less effective than in HT-29 cells. We compared the oncolytic effect of RCAd11pGFP on HT-29 and T84 cells and found that HT-29 cells showed 10 times greater susceptibility compared to T84 cells. Because the entry step of RCAd11pGFP infecting HT-29 is 10-fold more efficient than the same vector infecting T84 cells, oncolysis by the vector was actually comparable between T84 and HT-29 cells. These results may be attributable to the ability of RCAd11pGFP to selectively replicate and release virions from the colon carcinoma cells studied.

In xenograft nude mice, the tumor sizes of T84 and HT29 origin became significantly reduced after intratumor injection of RCAd11p or Ad11pwt viruses, relative to mock-injected tumors. The tumor killing effect on T84 cells was apparently more inhibited than on HT29 cells. These data are in accordance with our *in vitro* results, where RCAd11p and Ad11pwt showed a highly cytolytic effect in T84 and HT29 cells. To investigate cellular components, we used a polyclonal antibody to detect CEA family molecules and found that there was a correlation between the level of CEA family expression and the extent of virus replication and cell killing. CEA family molecules were expressed at their highest levels in T84 cells, followed by HT29 cells. In these two cell lines, the main CEA family molecules are CEACAM5 and CEACAM6 [31], [32], [33]. The exact function of CEA molecules still remains to be elucidated, not least to explain whether and how the CEA family molecules are involved in RCAd11pGFP replication, but we can conclude that the lytic activity of RCAd11p varied depending on the type of tumor cells investigated.

Although GFP expression in HCT-8 and LS174T cells was higher than in other colon carcinoma cells studied, the cytotoxicity and cell viability assays in HCT-8 were lowest than in other colon carcinoma cells during the late infection. This could be explained by the fact that adenovirus infection of cells can induce intracellular antiviral responses such as interferon type I, which can restrict virus replication, cell lysis, and virus spread. Interferon type I does not interfere with transgene expression from a non-replicating vector [34], perhaps due to the fact that adenovirus induces the interferon-regulatory factor during entry into the late phase of infection. In addition, HCT-8 cells may lack some element required for virus replication so that only limited amounts of virus particles are produced.

Studies using new, modified gene therapy vectors are reported every year, including the introduction of the E1A protein, introduction of a tissue-specific promoter, retargeting of the vector, or insertion of a new therapeutic gene. The results achieved are usually attributable to the modified vector constructs [13] [35]. In the present study, a comparison of the properties of RCAd11pGFP regarding its transduction ability and cell killing *in vitro* allowed us to rank the cell lines according to the highest lytic effect: the strongest effect was in T84, followed by HT-29 and then LS174T cells, with the lowest lytic effect being seen in HCT-8 cells. We recently demonstrated that the replication of RCAd11pGFP is independent of the level of p53 expression in metastatic prostate cells [36]. This property may be beneficial for colon carcinoma patients because about 50% of cancer carcinoma cells express a mutated p53 [37]; thus, gene therapy for colon carcinoma diseases based on the RCAd11p vector should be effective.

The transduction and oncolytic profiles of a replication-competent adenovirus 11p GFP vector (RCAd11pGFP) showed that this vector efficiently infects, productively replicates in, and induces oncolysis in the transplantable tumorigenic cell lines HT-29 and T84, which express high levels of CEA. We have demonstrated that despite the permissiveness of colon tumor cell lines to RCAd11pGFP and RCAd11pwt infection, variable cytotoxicity was observed *in vitro* and variable antitumor-specific inhibition was observed *in vivo*. Thus, the efficacy of the vector should be evaluated *in vitro* and *in vivo* using relevant tumors. The results also indicate that RCAd11pGFP is a potent and oncolytic vector in colon tumor cells, which express high levels of molecules of the CEA family.

Our results differ from those in a recent publication on the blockage of Ad5 trafficking in colon cancer cells by CEACAM6 [38]. Consequently, our results provide experimental evidence for the suitability of the biological properties of RCAd11p and for the feasibility of assessing the safety of RCAd11pGFP in preclinical gene therapy work aimed at controlling colon cancer.

## Materials and Methods

### Cell lines and culture conditions

A549, a human cell line from a lung oat cell carcinoma, was used as a control cell line because of its permissiveness to most human adenoviruses. All adenovirus stocks used in this study were produced in A549 cell cultures as previously described [25]. In addition, four colon carcinoma cell lines from different tissues and individuals were used: HT-29 cells, from colon adenocarcinoma (epithelial); LS174T and HCT-8 cells, also from colon adenocarcinoma (epithelial); and T84 cells, from colon carcinoma (epithelial-like) but derived from a lung metastasis. These four established human colon carcinoma cell lines were obtained from the American Type Culture Collection (Rockville, MD).

A549 cells were grown in Dulbecco's Modified Eagle's Medium (DMEM) supplemented with 5% fetal bovine serum (FBS) (HyClone). HT-29 cells were grown in McCoy's 5α medium with 10% FBS. LS174T cells were cultivated in DMEM with 10% FBS, 1-mM sodium pyruvate, and 1-mM non-essential amino acids. HCT-8 cells were grown in RPMI 1640 medium, 8% horse serum, and 1-mM sodium pyruvate. T84 cells were grown in a 1∶1 mixture of DMEM and F_12_ Ham's medium, with 8% FBS. All cells were cultivated at 37°C, and in addition, HT-29 and T84 cells were grown in an atmosphere of 5% CO_2_. As a negative control cell line, McCoy cells from a mouse fibroblast origin were grown in DMEM medium supplemented with 5% FBS.

### Adenovirus strains and virus preparation

Three species B adenoviruses [Ad11p (prototype strain Slobitski), Ad11a (BC34), and Ad35 (S763)] and one species C adenovirus [Ad5, (prototype strain Ad75)] were used for this study. Prototype Ad41 (strain Tak), a species F adenovirus, was also included in the study mainly due to its ability to bind to CAR. All viruses used were propagated in A549 cells and purified using CsCl gradients as described previously [39] [40].

### Vector construction

The RCAd11pGFP vector contains the entire Ad11pwt genome. In addition, the RCAd11pGFP possesses a GFP cassette directed by a CMV promoter. This entire cassette is inserted at the E1 region. Thus the RCAd11pGFP vector contains an entire viral genome with an added insertion. RCAd11p replication was initialled by virus own transcription elements. The biological properties of the RCAd11pGFP and the Ad11pwt are indistinguishable. RCAd11pGFP is a replication-competent adenovirus vector. The RCAd11pGFP vector was constructed as described, with some modification [41], and the key technologies used included homologous recombination in *E coli*.

### Viral infectivity assay

The colon carcinoma cells were seeded in growth medium on 12- or 24-well plates and cultivated for approximately 40 h. The medium was then replaced and the number of viable cells was counted. Infection at a concentration of one pg (about 3,600 virus particles) of RCAd11pGFP per cell was done at 37°C, starting with 1 h of shaking followed by incubation for 24 or 48 h. The cells were then fixed overnight in 2% paraformaldehyde (PFA) phosphate buffer, incubated with primary monoclonal antibody at 1∶200 (mab 8052 hexon antibody; Chemicon) for 1 h, and washed twice with the same buffer. They were then incubated for another hour with FITC-conjugated goat anti-mouse antibody and then assayed by flow cytometry (FACS) [40].

### Detection of cell-surface molecules

Five cell-surface molecules were measured in this study: (1) CAR, a major receptor for coxsackie B virus and for most adenoviruses except those of subgroup B; (2) CD46, which is a species B adenovirus receptor [24], [25], a glycoprotein present on the surfaces of human nucleated cells; (3) integrins αvβ3 and αvβ5, which can interact with the adenovirus penton base protein and promote virus internalization; and (4) CD80, a cell-surface glycoprotein, associated with T-cell maturation and suggested to be a primary receptor for species B adenoviruses [11]. For Flowcytometry (FACS) analysis, the following antibodies were used: mouse monoclonal anti-CAR (clone RmcB; Chemicon) diluted 1∶200; mouse anti-human CD46 monoclonal antibody (169-1-E4.3; Ancell) diluted 1∶200; rabbit anti-human polyclonal CEA IgG, 5 µg/µl, diluted 1∶200; anti-human integrin αvβ3 (MAB 1976; Chemicon) diluted 1∶200; anti-human integrin αvβ5 (MAB 1961; Chemicon) diluted 1∶200; anti-human (B7-1; Ancell) diluted 1∶50; and (5) antibody against CEA family molecules, which are highly expressed in many cancer types and in all colon carcinomas that have been examined [42].

Colon cancer cells were detached from a 24-well plate with PBS containing 0.05% EDTA, washed in PBS, and counted. They were then allowed to recover for 2 h in growth medium at 37°C. For each assay, 4×10^5^ cells were suspended in PBS containing 2% FBS and 0.01% NaN_3_. Monoclonal antibodies to the different receptors were added to the cell suspensions and incubated overnight on ice. Then, the cells were washed twice with the same buffer and incubated with secondary antibody for another hour on ice. For detection of CEA molecules, FITC-conjugated swine anti-rabbit antibodies (DAKO) were used. FITC-conjugated goat anti-mouse antibodies (Sigma) were used to detect the other surface molecules. After washing twice with PBS, the cells were analyzed by FACS.

### Detection of RCAd11p-mediated gene delivery

A predetermined number of cells of each line were seeded in 24-well plates to reach 4×10^5^ cells per well after 44 h of incubation. The cells were then infected with 18; 36; 180; 360; 1,100; 1,800; 3,600; 7,200 or 14,400 virus particles (vp) of RCAd11pGFP per cell. At 24 and 48 h post infection, the cells in each well were harvested by centrifugation at 800 rpm for 5 min and fixed in 0.5 ml of 2% PFA at 6°C. The fixed cells were washed twice in 1 ml of PBS and resuspended in 300 l of PBS. GFP expression was measured by FACS. The CPE and GFP expressions were also examined and documented photographically using a fluorescence microscope. All images were taken at 200× magnification.

### Cytolysis assay

The cells were seeded in 24-well plates and infected with 0.36; 3.6; 36; 360; 3,600 vp/cell of RCAd11pGFP as described above. The plates were stained every two days until day 12 post-infection (p.i.). To stain the cells with crystal violet, the cell medium was removed, and the cells were fixed with 4% PFA for 10 min at room temperature. They were then washed with PBS and incubated for 10 min with 1% crystal violet in 70% ethanol. After staining, the cells were rinsed three times with water and air-dried for photography.

### Toxicity assay

Cells (1×10^5^) were plated in 96-well plates and grown for 44 h. The cell medium was changed, and RCAd11pGFP was added in 10-fold dilutions as above. At 6 and 8 days p.i., 50 µl of XTT solution (Cell Proliferation Kit II, Roche) were added into 100 µl of cell medium and incubated for 2 h. Optical density (OD) was measured in a plate reader at wavelengths of 490 and 650 nm, and the OD at 650 nm was subtracted from that at 490 nm. Cell medium from uninfected cells was used as a negative control.

### Animal experiments

All animal protocols were reviewed and approved by the Umeå ethical board for the experimental animal and the Court of Appeal for Northern Sweden, The permit number is A96-07. BALB/c nude mice were purchased from Taconic (Ry, Denmark) and were used according to standard methods [35]. Because tumors induced by HT-29 cells grew much larger and faster than tumors induced by T84 cells in xenograft nude mice, 5×10^6^ HT-29 cells and 1×10^7^ T84 cells were subcutaneously injected into each side of nude mice. As each tumor grew to 75 mm^3^, 50 µg (1.78×10^11^ vp or 2.33×10^9^ moi; IP/PP: 1/72 vp) of RCAd11pGFP or Ad11pwt virus in 100 µl of PBS was intratumorally injected into each tumor, whereas injection with only PBS was used to mock-infect tumors. Tumor size was measured on a weekly basis and photographed.
